# Programmable Genome Editing Tools and their Regulation for Efficient Genome Engineering

**DOI:** 10.1016/j.csbj.2016.12.006

**Published:** 2017-01-12

**Authors:** Tuhin Kumar Guha, Alvan Wai, Georg Hausner

**Affiliations:** Department of Microbiology, University of Manitoba, Winnipeg, Manitoba R3T2N2, Canada

**Keywords:** Meganuclease, TALEN, Zinc finger nuclease, CRISPR/Cas9, Regulatory switch, Hammerhead ribozyme

## Abstract

Targeted genome editing has become a powerful genetic tool for studying gene function or for modifying genomes by correcting defective genes or introducing genes. A variety of reagents have been developed in recent years that can generate targeted double-stranded DNA cuts which can be repaired by the error-prone, non-homologous end joining repair system or via the homologous recombination-based double-strand break repair pathway provided a suitable template is available. These genome editing reagents require components for recognizing a specific DNA target site and for DNA-cleavage that generates the double-stranded break. In order to reduce potential toxic effects of genome editing reagents, it might be desirable to control the in vitro or in vivo activity of these reagents by incorporating regulatory switches that can reduce off-target activities and/or allow for these reagents to be turned on or off. This review will outline the various genome editing tools that are currently available and describe the strategies that have so far been employed for regulating these editing reagents. In addition, this review will examine potential regulatory switches/strategies that can be employed in the future in order to provide temporal control for these reagents.

## 1 Introduction

One of the challenges in biotechnology has been developing efficient and reliable ways to make targeted changes within the genome of cells. Traditional approaches of mutagenesis utilizing chemical agents or transposons can require extensive screening in order to recover desired mutations [1], [2], [3], [4], [5], [6]. Genome editing strategies using double-stranded (ds) DNA viral vectors in differentiated human cells and RNA interference (RNAi) mediated targeted gene knockdown approaches also have some pitfalls [7], [8], [9], [10]. For example, the protein composition of the viral capsid can be potentially immunogenic. Moreover, abnormal gene expression along with insertional mutagenesis may be triggered if there are random mutations in the viral sequences. On the other hand, the use of exogenously introduced dsRNA in RNAi technology can disrupt the “homeostasis” of the cellular machinery involved in gene silencing. Currently, the most popular genome engineering techniques apply DNA-cutting enzymes/complexes that generate targeted double-strand cuts [11], [12], [13], which are repaired by the host cells by either the error-prone, non-homologous end joining repair system (NHEJ), or the homologous recombination-based double-strand break repair pathway (HDR) [14], [15], [16], [17], [18]. The most frequent application of these endonuclease-based tools is the study of gene function through the inactivation of the target gene [19], [20], [21]. In addition, by providing a repair template, these systems allow for gene replacement strategies by taking advantage of the host cell's dsDNA break homologous repair system [22], [23], [24]. These new methods have tremendous potential towards the development of more accurate cellular and humanized laboratory animal models for various pathological conditions [25], [26]. Moreover, these endonuclease-based genetic engineering techniques are being developed as therapeutic agents to cure human monogenic diseases [27], [28], [29], [30], [31]. Genome editing tools have far-reaching implications in the agricultural sector and in their potential of curbing pest populations, such as malaria insect vectors, or invasive species, such as cane toads and carps [32], [33], [34], [35], [36]. The latter applications are achieved in promoting the ‘gene drive’ of an introduced genetic element (such as a meganuclease) within an interbreeding population that can distort sex ratios (daughterless generations), or target genes related to fertility or pathogenicity [37], [38], [39], [40], [41].

Genome editing tools include meganucleases (MNs) [42], [43], [44], [45], zinc finger nucleases (ZFNs) [46], [47], [48], [49], transcription activator-like effector nucleases (TALENs) [50], [51], [52], [53], clustered regularly interspaced short palindromic repeat (CRISPR)-associated nuclease Cas9 [54], [55], [56], and targetrons [57], [58], [59], [60], [61], [62], [63]. All of them can achieve precise genetic modifications by inducing targeted DNA double-strand breaks (DSBs). Depending on the cell cycle stage, as well as the presence or absence of a repair template with homologous terminal regions, the DSB may then be repaired by either NHEJ or HDR [64], [65], [66], [67], [68]. NHEJ can result in frameshift mutations that usually lead to gene disruption or gene knockout and/or the production of nonfunctional truncated proteins [69], [70], [71]; one exception being when a frameshift mutation was introduced to correct a defective coding sequence in the dystrophin gene [72], [73]. In contrast, when single- or double-stranded DNA templates with homologous sequences that correspond to sequences flanking the break site are introduced within the cell, the lesion may be repaired using the HDR machinery [74], [75].

One crucial concern when applying these genetic editing tools is the potential of cleavage at non-targeted sites. This event can be lethal or generate undesirable mutations resulting in the requirement of extensive screening in order to identify cells with the desired site-specific modifications. Many excellent reviews are available with regards to the above listed genome editing tools [13], [21], [42], [44], [45], [76], [77], [78], [79], [80], [81], [82], [83], [84], [85], [86], [87]. Therefore, this review will provide only a brief overview of the current genome editing tools and note any modifications made within recent years. The major focus in this review is to examine the efforts that have been made in the development of programmable, endonuclease-based platforms and various molecular switches that could be employed for the temporal regulation of these DNA-cutting enzymes in order to reduce off-target activities. The term “programmable” refers to the ability to engineer the nuclease-based platforms for recognizing various target sites (i.e. target specificity) in the genome.

## 2 Genome Editing Reagents

**Fig. 1:**
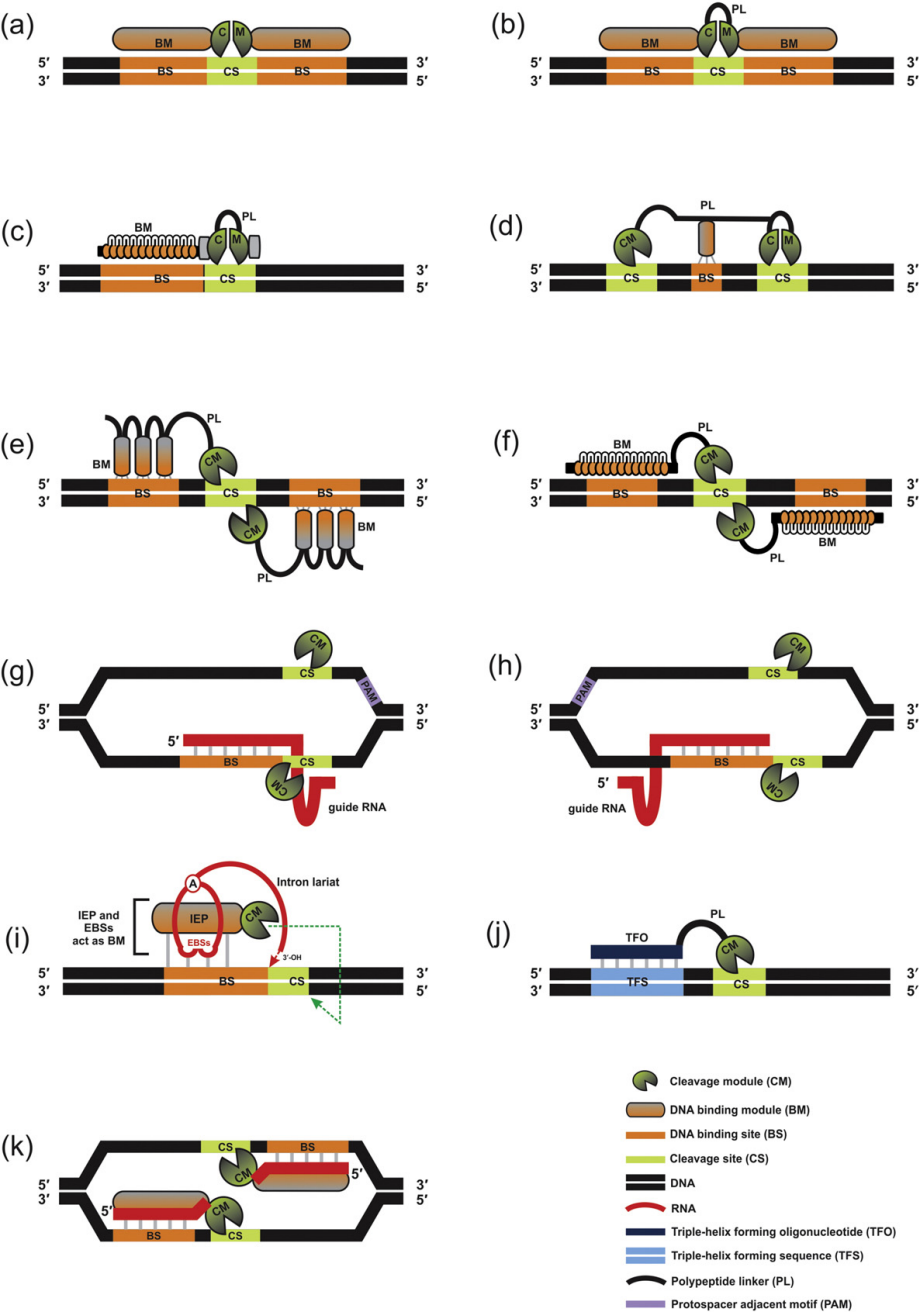
Examples of programmable genome editing tools. (a) Single-motif LAGLIDADG homing endonucleases, (b) double-motif LAGLIDADG homing endonucleases, (c) megaTAL, (d) MegaTev, (e) zinc-finger nucleases (ZFN), (f) transcription activator-like effector nucleases (TALENs), clustered regularly interspaced short palindromic repeats (CRISPR)/CRISPR-associated proteins (Cas) systems using (g) Cas9 or (h) Cpf1, (i) targetrons, (j) triplex-forming oligonucleotide (TFO) nucleases, and (k) structure-guided nucleases (SGNs). EBS = exon-binding site; IEP = intron-encoded protein. The nuclease domain of FokI is used to engineer ZNFs, TALENs, and SGNs. Elements of this figure have been adapted from Hafez et al. [44] NRC Research Press License number: 3981970186164.

In general, genome editing tools using DSB nuclease-driven reactions (Fig. 1) can be divided into two groups. The first group consists of MNs, ZFNs and TALENs, which achieve sequence-specific DNA-binding via protein-DNA interactions [13], [42]. The second group is comprised of two sub-groups: (i) CRISPR/Cas9 and targetrons, which are RNA-guided systems [56], [57] and (ii) peptide nucleic acids (PNAs), triplex-forming oligonucleotides (TFOs), and structure-guided endonucleases (SGNs), which are DNA-based-guided systems [88], [89], [90], [91], [92]. A generalized comparison for the more commonly used genome engineering tools is presented in Table 1.

Meganucleases, or homing endonucleases (HEases; Fig. 1a,b), are highly site-specific dsDNA endonucleases that can be reengineered to expand their target site repertoires using various strategies, such as computational structure-based design, domain swapping, combined with yeast surface display for efficient detection of HEases with desired sequence specificities [93], [94], [95], [96], [97], [98]. The LAGLIDADG family of MNs have been extensively studied and applied as genome editing tools [43], [44], [45], [99], [100], [101]. Unless otherwise mentioned, we are referring to LAGLIDADG enzymes as MNs for simplicity. One essential drawback for this class of enzyme is its non-modular configuration. The DNA recognition and cleavage functions can be, in part, intertwined in a single protein domain. Therefore, engineering of MNs has been challenging [45], [76] and has resulted in the development of other editing tools. However, a recent study suggests that there are multiple points across the LAGLIDADG protein that can be involved in holding metal ions in suitable positions to facility cleavage [102]. This finding along with technologies, such as yeast surface display-SELEX, still hold promise for MNs to be engineered more efficiently in the near future [97]. Moreover, a single-chain modular nuclease architecture, termed ‘megaTAL’ (Fig. 1c), was designed in which the DNA-binding region of a transcription activator-like (TAL) effector is appended to a site-specific MN for cleaving a desired genomic target site [103]. The latter synthetic version of a MN provides a modular design, separating the endonuclease and DNA binding activities. Therapeutic applications that demand precision with regards to gene modification activity can be addressed by these engineered variants of MNs, as they are considered to be highly target-specific ‘molecular scissors’ [45]. MNs are also in demand as components of vector/cloning systems (e.g. HomeRun vector assembly system) and synthetic biology applications (e.g. iBrick) that require rare-cutting enzymes [104], [105].

Even though the NHEJ pathway is usually exploited to introduce mutations at the DSBs within the genome [15], [106], sometimes, DSBs possess compatible “sticky” ends that can be repaired without any introduced mutation [107]. Recently, the ‘MegaTev’ (Fig. 1d) architecture has been generated which involves fusion of the DNA-binding and cutting domain from a meganuclease (Mega, I-OnuI) with another nuclease domain derived from the GIY-YIG HEase (Tev, I-TevI). This protein was designed to position the two cutting domains ~ 30 bp apart on the DNA substrate and generate two DSBs with non-compatible single-stranded overhangs for more efficient gene disruption [108]. More recently, similar to the MegaTev concept, Wolfs et al.have designed another dual nuclease, in which the Tev endonuclease domain is attached to the Cas9 nuclease domain, known as TevCas9 [109]. This hybrid nuclease, when introduced within human embryonic kidney cells (HEK293) along with appropriate guide RNAs, has been shown to delete 33 to 36 bp of the target site, thereby creating two non-compatible DNA breaks at moderately higher frequencies (40%). Therefore, this newly designed dual active endonuclease also promises to favor genome editing events (i.e. introduce mutations) by avoiding the creation of compatible “sticky” ends which lead to a failed attempt of genome editing [109].

More recently developed genome editing tools try to be more flexible with regards to retargeting the reagent to different sequences by having a modular design: a DNA-cutting domain (that can be non-specific) and a distinct programmable DNA-binding domain. The ZFNs are artificial endonucleases that have been generated by combining a small zinc finger (ZF; ~ 30 amino acids) DNA-binding/recognition domain (Cys_2_His_2_) to a type IIS nonspecific DNA-cleavage domain from the FokI restriction enzyme (Fig. 1e). However, the cleavage activity of the FokI endonuclease demands dimerization [46], [110]. As a ZF module recognizes a 3 bp sequence, there is a requirement for multiple fingers in each ZFN monomer for recognizing and binding to longer DNA target sequences [46]. In the past, using structure-based design, two ZFN variants were engineered that efficiently cleaved DNA only when paired as a heterodimer, thereby providing a potential avenue for improving the specificity of ZFNs as gene modification reagents [111]. In a different structure-based study, using 3D protein modeling and energy calculations through computer-based softwares, researchers have identified potential residues within the FokI dimer interface that are responsible for ZFN dimerization [112]. These newly designed ZFNs were considered significantly less genotoxic (i.e. cleavage at on-target sites) in the cell-based recombination studies because the homodimerization could be prevented by lowering the dimerization energy, hence prevent activation of the dimeric FokI [112].

Recently, ZFNs have been used as a potent antiviral therapy in the inactivation of specific coreceptors, thereby protecting cells from the viral entry in order to establish infection [113]. Even though ZFNs showed impressive results in modifying the HIV CCR5 coreceptor surface protein in the autologous CD4 T lymphocytes of persons infected with HIV [114], there is still the risk of cleavage at ectopic sites due to the modular architecture of ZFNs and the non-specific nature of FokI [49], [115].

Apart from implementing ZFNs as genome editing tools [48], [49], recently, the artificial zinc-finger protein (AZP)-staphylococcal nuclease (SNase) hybrid was designed (AZP-SNase) for potential antiviral therapies. This artificial nuclease can bind and cleave a specific origin of replication sequence of the human papillomavirus type 18 (HPV-18) thereby inhibiting viral replication in mammalian cells [116]. However, one disadvantage of this reagent is that the SNase has been shown to cleave both single and double-stranded RNA as well as the host DNA (single or double-stranded). Further modification involving switching of the SNase moiety in the AZP-SNase to the single-chain FokI dimer (scFokI) cleaved the viral DNA. Therefore, this newly designed hybrid ZFN is expected to serve as a novel antiviral reagent for inactivating human DNA viruses with fewer side effects [117].

TALENs are artificial endonucleases (Fig. 1f) designed by fusing the DNA-binding domain (multiples of nearly identical repeats each comprised of ~ 34 amino acids) obtained from TAL (transcription activator-like) effector (TALE) protein to the cleavage domain of the FokI endonuclease [118]. Each TALE repeat independently recognizes its corresponding nucleotide (nt) base with two variable residues [termed the repeat variable di-residues (RVDs)] such that the repeats linearly represent the nucleotide sequence of the binding site. Despite the tolerance to mismatches of longer TALENs in vitro, they seem to have higher genome editing activity and considered less genotoxic than ZFNs [119], [120], [121], [122], [123]. TALENs can be redesigned to bind user-defined sequences by simply joining appropriate repeat units. Like ZFNs, TALENs are dimeric in nature; this necessitates the design of two independent DNA-binding modules to target a single sequence. One advantage of the requirement for dimerization is enhanced specificity over monomeric enzymes [50], [51]. Although the FokI enzyme is useful in terms of flexibility in the choice of various target sites, its nonspecific activity also increases the probability for more frequent cleavage at off-target sites in the genome [124]. As an alternative approach to the FokI-based architecture, monomeric Tev-TALE nucleases (Tev-mTALENs) were created. Here, the sequence-specific, monomeric nuclease domain from the I-TevI HEase is fused with TALEs. Thus, only a single DNA-binding module is needed to target a sequence for cleavage. However, the use of a domain with predetermined recognition requirements, like TevI, significantly limits the range of genomic targets [124].

Components derived from the bacterial “immunity” system, CRISPR locus and the Cas9 nonspecific endonuclease (CRISPR/Cas9), form a novel RNA-guided endonuclease (RGEN; Fig. 1g) for precise and efficient gene targeting [125], [126], [127], [128]. The uniqueness of this platform is based simply on designing guide RNAs (gRNAs) essentially serving as CRISPR RNAs (crRNAs) that are bound by the Cas9 nuclease. Initially, the gRNAs were expressed separately as trans-activating CRISPR RNA (tracrRNA) and the “user-designed” crRNA sequence, both of which are chemically synthesized for the effective targeting and cleavage of a sequence within the gene of interest [129]. More commonly, for simplicity, both the crRNA and tracrRNA are expressed as a single construct known as single guide RNA (sgRNA) [55]. Cas9, however, does not require any engineering for retargeting. Complementary base pairing allows a segment of the gRNA sequence (~ 18–20 nt) to hybridize with the targeted DNA sequence and thus docking of the Cas9 nuclease at that location. The H–N–H and the RuvC nuclease domain of the Cas9 cleave both DNA strands to create DSBs 3 bp upstream (5′) of the protospacer adjacent motif (PAM) sequence. The PAM sequence is specific to each Cas9 nuclease obtained from different bacterial species [130], [131]. Therefore, different sources for Cas9 have to be explored with regards to optimizing this system to a wide range of eukaryotes/target sequences. Eventually, by designing various gRNAs, this system can be utilized for targeted mutagenesis by inducing the NHEJ pathway or it can be applied to repair or replace alleles by utilizing the cellular HDR repair mechanism with the presence of a user-provided DNA corrective template.

A modified version of the RNA-guided Cas9 has been developed that allows for “targeting” regulatory sequences and manipulating gene expression. For this purpose, a nuclease-deficient version of Cas9 protein has been generated by mutating positions H840A in the H-N-H domain and D10A in the RuvC domain [132]. This variant is commonly known as “dead” Cas9 or dCas9. However, the DNA-binding characteristic remains unaffected for this modified protein [55], [133]. Therefore, gene silencing (referred to as CRISPR interference or CRISPRi) or gene activation can be made possible by fusing dCas9 with various effector domains [134], [135], [136], [137], [138], [139], [140].

In a recent study, GCaMP (a calcium-sensitive modified GFP) fluorescence signals were monitored in induced pluripotent stem cells (iPSCs) to determine if CRISPRi, based on the RNA-guided dCas9 being targeted to bind to a specific promoter sequence, can knock down GCaMP expression and whether removal of doxycycline [tetracycline (Tet) derivative] from the culture reversed its expression. Expression of the CRISPRi components are under the control of the Tet-response element (TRE), thus doxycycline acts as an inducer for the regulatory protein that interacts with the TRE. The researchers found that GCaMP expression was downregulated by 98% after addition of doxycycline for 7 days. However, the expression was completely restored after removing doxycycline for 14 days [141]. This proof of principle study demonstrated that reversible RNA interference is possible with regulated versions of dCas9 and this might become a powerful alternative to RNAi, which can be applied to knock down expression of a gene but cannot be reversed. Furthermore, dCas9 has been repurposed as a visualization tool. For example, Enhanced Green Fluorescent Protein (EGFP), when fused with dCas9, enabled visualization of both repetitive and nonrepetitive DNA sequences [142]. Recently, the dCas9 has also been used as a building block for RNA-guided FokI nucleases, thereby dCas9 also has applications in genome editing. Here, the dCas9 and its sgRNA has been recruited as a DNA-binding module that is coupled with FokI, which serves as the nuclease component [143]. This reagent requires dimerization that is brought about by the FokI-dCas9 fusion proteins being recruited to sequences adjacent to the target site by two different gRNAs.

Multiplex editing is possible with CRISPR/Cas9 [144], [145], [146], [147], [148] and the PAM requirements of Cas9 do not place much of a limitation on target choice because PAMs are quite short sequences [125], [131]. However, the risk of off-target activities exists [149], [150]. Henceforth, paired Cas9 nickases and gRNA modifications, like truncated gRNA (tru-gRNA), have been constructed and have shown promising results with regards to reducing off-target activities [151], [152], [153], [154].

Another recent innovation is the isolation of the novel CRISPR protein, Cpf1, a non-Cas9 CRISPR nuclease (Fig. 1h). Cpf1 has been shown to generate staggered double-strand breaks with “sticky ends” at targeted sites, which is not the case for Cas9 proteins [155]. The generation of sticky ends and the programmability of the CRISPR/Cpf1 endonuclease system make this reagent very suitable for developing DNA assembly strategies (e.g. C-Brick) [156]. Cpf1 requires a T-rich PAM sequence, making this reagent suitable for targeting T-rich segments within genomes [155], [157]. Moreover, Cpf1 seems to have inherently higher specificity than currently available forms of Cas9 [158], [159]. A variant of Cas9, recently described from *Staphylococcus aureus*, is considerably smaller (by 1 kb) compared to other bacterial Cas9 proteins. This represents an improvement as it allows for the design of more compact vector systems that are more easily accommodated within the more efficient viral-based delivery systems for in vivo or ex vivo applications [160].

Development of the “enhanced specificity” SpCas9 (eSpCas9) through structure-guided protein engineering has shown a dramatic decrease in off-target indel (insertions-deletions) formation, thereby contributed towards a significant improvement over the *Streptococcus pyogenes* Cas9 (SpCas9) enzyme [161]. In this study several SpCas9 mutants were designed by substituting 32 positively-charged residues, which are responsible for recognizing the nucleotide groove, with individual alanine moiety. Then after, using a previously validated guide sequence, these single amino acid SpCas9 mutants were tested for specificity by targeting them to the *EMX1* target site in human embryonic kidney (HEK) cells. With these improved versions, the specificity of indel formation at the target sites has been shown to be improved by a factor of 2 to 5 [161].

Usually, genome editing tools introduce dsDNA breaks at a target locus. However, a recent study has shown that one could bypass the need for dsDNA backbone cleavage and the required introduction of a donor template for genome editing. Strategies are being developed that harness enzymes that can edit DNA sequences by chemically modifying nucleotide bases [162]. For example, it has been shown that fusing rAPOBEC1 cytidine deaminase [163], which showed the highest deaminase activity among the four different deaminase enzymes tested, to the amino-terminus of dCas9, does not affect the deaminase activity. Therefore, ‘base editing’ using cytidine deaminase may be an alternative new approach to genome editing that enables irreversible conversion of one target DNA base into another. In that study, direct conversion of cytidine to uridine in a programmable manner has been shown to be possible with the help of a guide RNA [162]. However, how would uridine, which is one of the building blocks of RNA, be tolerated within the DNA sequence is questionable. Usually, cytidine deaminases use RNA as the substrate and, interestingly, a few of them have been reported to work on single stranded (ss) DNA. Fortuitously, when dCas9-target DNA complex is formed, the displaced DNA strands are separated to form the ‘R-loop’ complex whereby both the strands are separated. This conformation might serve as an efficient substrate for this programmable conversion of cytidine to uridine in DNA. One of the major challenges of this technique is that it is unable to perform precise base editing, in particular when multiple cytidines are present in close proximity, i.e. the spreading of base modification to neighboring cytidine occurs [162].

Although not a form of genome editing, another noteworthy development is the nuclease-inactive *S. pyogenes* RNA targeting CRISPR/Cas9 (RCas9) protein that is conjugated with the green fluorescent protein [164]. This reagent has been engineered to bind to RNA with the aid of a sgRNA strands. The sgRNA allows for the system to be programmable, thereby allowing for endogenous RNA tracking in living cells [164].

The targetron (Fig. 1i) is a ribonucleoprotein particle (RNP) that consists of an engineered group II intron RNA lariat molecule and a multidomain group II intron-encoded protein [i.e. reverse transcriptase (RT)] which has been used for mutagenesis of bacterial genes [57], [58], [59], [60]. The strategy is based on group II retrohoming where the intron lariat recognizes its native DNA target site by the presence of an “exon-binding sequence” (EBS) that can base pair with a corresponding “intron-binding” sequence (IBS) present within the targeted gene/site. These “EBS/IBS” interactions require homology for about ~ 14 bp [61]. This RNA-guided endonuclease system has shown potential for highly site-specific retro-targeting (mutagenesis by insertional mutations) of genes in prokaryotes by simply reprogramming the intron EBS to match target sequences within targeted genes [62]. Compromised activity is observed in eukaryotes, such as mammalian systems, due to suboptimal codon usage, translational repression of the RT, nonsense-mediated decay (NMD) of group II intron-containing RNAs, and suboptimal magnesium ion (Mg^+ 2^) concentrations [78]. In addition, the entry of the targeting RNA, in the form of an RNP, into the nucleus or chromatin still remains the major obstacle for applications of targetrons among eukaryotes [63].

Synthetic molecules such as peptide nucleic acid (PNA) oligomers [88] and triplex-forming oligonucleotide (TFO; Fig. 1j) [165] have been developed as potential alternatives to the above outlined genome editing reagents. The strategy is to develop programmable DNA-binding modules that can be coupled to DNA-cutting domains. Although their use, so far, has been limited, they do offer some advantages that are worth mentioning. For example, PNAs have higher binding strength compared to oligonucleotides [166]. Therefore, designing long PNA oligomers for use in DNA-binding is not a prerequisite. This is in contrast with the targetron and the CRISPR/Cas systems, which usually require DNA-binding modules of 14–22 bases for efficient recognition and DNA-binding [55], [57]. Moreover, PNAs can tolerate a wide pH range and are not easily recognized by either nucleases or proteases [167]. Also, improvements regarding the delivery within the cytoplasm have been made when different cell-penetrating peptides were coupled to PNAs by covalent bonds [167]. The TFO nucleases are sequence-specific type II restriction enzyme-TFO conjugates [165]. Instead of a protein-based DNA-binding domain, as seen in MNs, ZFNs, or TALENs, these DNA-binding oligonucleotides can be engineered to cater to various DNA target sites. However, the DNA-cutting components of TFO nucleases are activated by Mg^+ 2^ ions, and thus cleavage activity might be triggered before the RE-TFO conjugate assembles on the intended target site [168], [169]. There are also versions of TFOs that operate as dimers and utilize FokI as the nuclease domain [170]. Interestingly, both PNAs and TFOs can be also designed to target RNA duplexes forming RNA triplexes, which may have potential application in gene regulation [171].

Another new entry among potential genome editing tools is the structure-guided endonuclease (SGN; Fig. 1k), which is composed of the flap endonuclease-1 (FEN-1) attached to the FokI nuclease domain [90]. In eukaryotes, FEN-1 is involved in DNA repair and DNA replication that involves the removal of 5′ overhanging flaps and in processing the 5′ ends of Okazaki fragments in lagging strand DNA synthesis [90], [91]. The engineered SGN complex operates as a dimer and is guided to a target site by two single-stranded guide-DNAs (gDNAs, 20 to 60 nts). The gDNAs are designed to have a single-base mismatch at the 3′ end; i.e. a 3′ “flap” structure forms once these oligonucleotides have bound to their targets. The FEN-1 component of the SGN recognizes a 3′ “flap” structure and is recruited to this position. Thereafter, the Fok1 dimer will form and cleave the target DNA strands. This approach has been successfully demonstrated in zebrafish embryos and therefore has potential for genome editing among the metazoans [92]. It was noted that SGN can generate large deletions at the cut site, probably due to the combined activities of the FEN-1 and FokI nuclease domains. This might be an advantage when the goal is to achieve gene disruptions [92].

The CRISPR/Cas system has definitely expedited biological research with regards to genome editing. However, recent work involving the Argonaute family of proteins from *Natronobacterium gregoryi* hints at the possibility of another option for genome editing in mammalian cells [172]. Gao et al. (2016) noted that NgAgo (*N. gregoryi* Argonaute) with the aid of DNA oligonucleotides can be programmed for site-specific targeting. The 5′ phosphorylated single-stranded guide DNA (gDNA) is usually 24 nt long sequence, and when bound to the NgAgo protein it is sufficient to create a DSB at the corresponding DNA target site. This system has the potential to edit GC-rich regions within the genome and does not have a PAM sequence requirement, thus allowing for a wider range of genomic targets [172]. However, this work is currently under scrutiny as other groups noted that the work was not reproducible in their laboratories [173]. Therefore, considerable efforts may yet be required to demonstrate the promised utility of the NgAgo-gDNA based system for genome editing.

In addition to the above genome editing reagents, site-specific recombinases have been shown to work efficiently as genome engineering tools in mammalian cells [174], [175]. These recombinases have been mostly derived from the bacteriophages, such as the Cre resolvase from the P1 phage of *Escherichia* *coli* and phiC31 integrase from a phage of *Streptomyces* sp. [176], [177]. These recombinases are highly site-specific and recognize long DNA binding sites of 34 bp. Unlike the above genome editing tools, these enzymes can process DNA strand exchange in a “cut and paste” fashion without creating any free DSB. This means that the complete recombination happens immediately in a concerted manner within the “all-in-one” recombinase enzyme complex, without being assisted by other cellular enzymes [178]. Typically, the phiC31 integrase assists in a unidirectional recombination between two different attachment (*att*) sites (*attB* and *attP*), resulting in the integration of a plasmid or any other DNA fragment quite precisely within the chromosome [179]. Fortuitously, along with *att* sites, the human genome and other larger genomes contain pseudo-*attP* sites [180]. With regards to human gene therapy applications it was noted that a variant of the phiC31 integrase (a 613-amino acid protein) can recognize these pseudo-*attP* sites, and thereby is able to insert DNA molecules, such as therapeutic genes or plasmids at preferred sequences within the mammalian genomes [181].

## 3 Current Regulatable DNA-cutting Enzymes

**Fig. 2:**
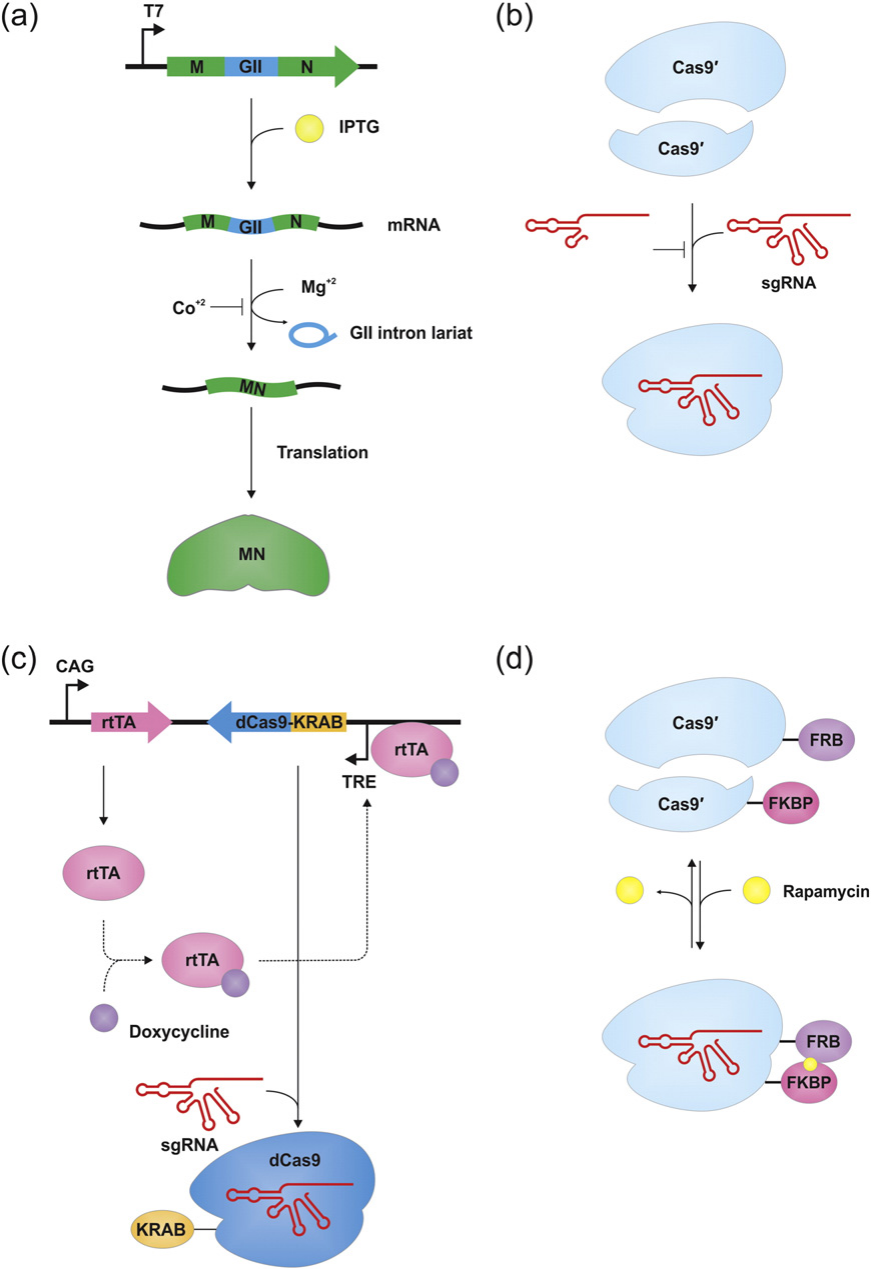

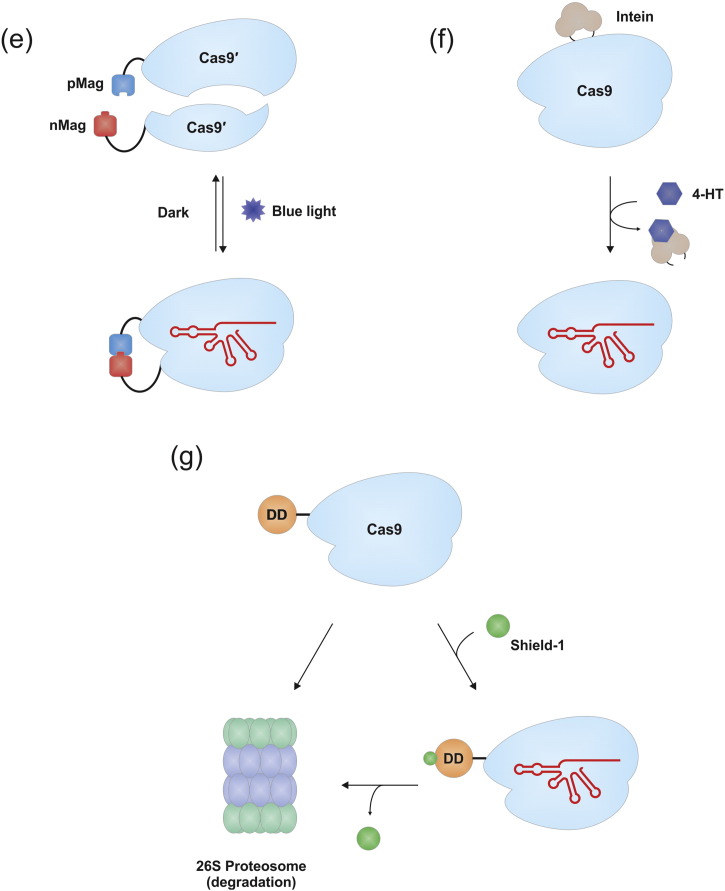
Strategies used to modulate Cas9 activity. (a) Group II intron (GII)-based switch, (b) separating Cas9 into two peptides, termed split-Cas9, (c) Tetracycline-inducible and reversible expression system, and (d) ligand-dependent dimerization of split-Cas9. Note: the strategy illustrated in (a) is based on the original study conducted by Guha and Hausner [185] on modulating expression of a meganuclease, not Cas9. A similar case is observed in (c), where Mandegar et al. [141] modulated the expression of dCas9, not Cas9. In both cases, a similar approach might also be possible with Cas9. (e) Light-dependent dimerization of split-Cas9, termed photoactivatable Cas9 (paCas9), (f) intein-Cas9, which are activated by splicing of a ligand-dependent intein, (g) and unstable destabilizing domain-Cas9 (DD-Cas9) fusions, which are degraded unless provided with the ligand, Shield1. Abbreviations: CAG = cytomegalovirus early enhancer/chicken β-actin promoter; Cas9 = clustered regularly interspaced short palindromic repeat (CRISPR)-associated protein 9; Cas9′ = partial Cas9; dCas9 = dead Cas9; FKBP = FK506 binding protein; FRB = FKBP-rapamycin binding; IPTG = isopropyl β-D-1-thiogalactopyranoside; KRAB = Krüppel-associated box; MN = meganuclease; mRNA = messenger RNA; rtTA = reverse tetracycline-controlled transcriptional activator; sgRNA = single-guide RNA; TRE = tetracycline response element; T7 = T7 RNA polymerase promoter; 4-HT = 4-hydroxytamoxifen; DD = destabilizing domain; nMag = negative Magnet; pMag = positive Magnet; sgRNA = single-guide ribonucleic acid. See text for more details.

In some instances, such as in vivo or ex vivo gene targeting, temporal regulation of endonuclease activity might be desirable in order to minimize nonspecific activity of the DNA-cutting enzymes (Fig. 2). DNA-cutting enzymes ultimately can have mutagenic and/or toxic side effects if they go off-target. Previously, a reversible redox switch was developed that controlled the endonuclease activity of PI-SceI in vitro [182]. Here two cysteine amino acid residue pairs were inserted into the HEase DNA-binding loops to allow for disulfide bond formation (oxidizing condition) that locks the endonuclease into a nonproductive conformation. This can be reversed by reducing conditions that result in the breakage of the disulfide bond, thereby yielding an active conformation of the protein. Since the inside of cells have reducing environments, this approach is not practical for activating the enzyme during in vivo applications [182].

Recently, it was shown that expression of an active MN, I-CthI [183], can be controlled, or at least attenuated, by the splicing activity of autocatalytic group II intron sequences (Fig. 2a) [184], [185]. The expression and activity of I-CthI HEase was modulated in *E. coli* by inserting ribozyme type introns (group IIA and IIB intron sequences), that lack open reading frames (ORFs), separately into the MN ORF, where splicing of these introns could be stimulated by the addition of 5–10 mM Mg^+ 2^ and antagonized by the addition of 10 μM cobalt ions (Co^+ 2^) in the bacterial growth media. Group II intron sequences are readily available [186], [187], [188], deposited in various databases, and these sequences could be coopted as regulatory switches [184] and unlike previous attempts to control MN activity via in vitro redox switches [182], in vivo regulation of endonuclease activity utilizing group II introns is possible [184], [185]. In the future, with regards to group II intron-based “switches”, one could achieve even tighter control by utilizing trans-splicing group II introns. Trans-splicing group II introns (or fragmented group II introns) have been noted in organellar genomes but it is unknown if these types of introns can function in *E. coli* [189], [190]. However, it has been shown that the Ll.LtrB group II intron (including a version where the ORF was deleted) from the Gram-positive bacterium *Lactococcus lactis* can splice in *trans* when fragmented at various locations throughout its structure [191]. Therefore, a MN ORF could be split and encoded by two compatible plasmids carrying different selectable markers and different promoters. One construct can bear the amino-terminal part of the HEase ORF plus the 5′ segment of a group II intron sequence and the other construct can carry the 3′ segment of group II intron sequence plus the carboxyl-terminal part of the MN ORF. Upon expression, these two RNAs can assemble via the intron segments into a tertiary structure that promotes trans-splicing of the intron sequences. Thus, the exons get ligated together to produce a functional MN transcript. Even though group II intron-based “molecular switches” have been shown to work quite efficiently in bacterial systems, they may only have limited applications in eukaryotes. Compromised activity of group II intron splicing and retrohoming in nuclear environments has been noted to be due to the suboptimal intracellular Mg^+ 2^ concentrations [78]. In addition, intron-containing transcripts are subjected to NMD and translational repression [63]. However, recent work by Lambowitz's group showed progress towards developing a group II intron expression system that can circumvent expression/splicing barriers. They have shown that retrohoming into chromosomal target sites in human cells at appreciable frequencies is possible when Mg^+ 2^ salts are added to the culture medium [192], [193]. Through genetic selections and deep sequencing techniques, they also identified several group II intron RNA mutations in the catalytic core domain V (DV) that partially rescued retrohoming in Mg^+ 2^-deficient *E. coli* [194] and in human cells at low Mg^+ 2^ concentrations [78]. Their findings have implications in terms of demonstrating the feasibility of selecting various group II intron variants that function more efficiently at low Mg^+ 2^ concentrations. Also, recent characterization of group II introns that are less dependent on Mg^+ 2^ may offer new impetus on the utilization of group II ribozyme-based switches in eukaryotic systems [194]. For now, one can foresee the application of group II intron sequences as agents that allow for inducible genome editing in cell types that are suited towards supporting the splicing of these elements.

It has been documented that constitutive expression of the Cas9 enzyme is one of the problems limiting the use of the CRISPR/Cas9 systems. Constitutive expression or high dosage of the Cas9 can lead to an increase in indel frequencies at off-target sites thereby initiating a DNA damage response [79], [149]. However, another study showed that the Cas9 enzyme alone is quite well tolerated, particularly in mice. Therefore, viable mouse models expressing Cas9 constitutively do exist [145]. Apart from transient delivery of purified Cas9:sgRNA complex into cellular environments [195], [196] and regulating expression through the use of inducible promoters [197], [198], several methods have been developed with regards to addressing the regulation of this enzyme. Initial attempts to separate or split the Cas9 protein into two fragments have been successful. The Cas9 protein was separated in two polypeptides, one expressing the nuclease lobe and the other expressing the α-helical lobe of the enzyme (Fig. 2b) [199]. The two modules interacted and combined only in the presence of a sgRNA, thereby restoring the activity of a full-length Cas9. The enzymatic activity of the holoenzyme formed from two peptide components was shown to be no different from that of the native Cas9 and therefore remained effective for genome editing in human cells when full-length sgRNAs were used. However, shortening or modifying the sgRNAs, particularly removing the hairpins 1 and 2 from the 3′ end of the sgRNA structure rendered the protein modules in a separated, inactive conformation [199].

As an alternative to the above, there are versions of the Cas9 enzyme that are split into two components that can reconstitute into an active Cas9-gRNA complex by the addition of chemical signals, such as doxycycline and rapamycin (Fig. 2c,d) [200], [201]. Reversibility of these systems can be achieved upon the withdrawal of these ligands. Even though inducible methods based on plasmid constructs that included various regulatory elements that can be modulated to determine the expression of various CRISPR components have been used for generating conditional gene knockouts and reducing off-target effects during genome modification, one important concern still lurks regarding the adversities of these chemicals (i.e. inductants, ligands). Also ligands required for components to assemble at the protein level also may be of concern with regards to side effects on the cells, and this may limit their in vivo or ex vivo applications. For example, inducing the dimerization domains with rapamycin can perturb the endogenous mammalian target of rapamycin complex 1 (mTOR1) pathway leading to undesirable biological effects [200], [202]. However, the possibility of building an array of other inducible split-Cas9 enzymes that utilizes the same concept but depend on other chemical-sensing domains, such as abscisic acid or gibberellin-sensing domains may be an effective alternative in terms of toxicity. The utility of these domains towards induction, however, needs to be tested before they can be introduced in animal or plant cells.

Light can be controlled both temporally (microseconds) and spatially (microns) and is noninvasive to biological systems (Fig. 2e) [203], [204], [205]. Therefore, regulating the activity of DNA-cutting enzymes using light as a trigger may be an alternative to the above described approaches. Recently, this concept was applied to the genome editing of human cells by engineering a photoactivatable Cas9 (paCas9) that allows for optogenetic/light control of the CRISPR/Cas9 system [206]. Briefly, paCas9 consists of split-Cas9 fragments, each appended to photoinducible dimerization domains named “Magnets”. Both positive (pMag) and negative (nMag) “Magnets” are light inducible dimerization proteins (~ 150 amino acids each), which heterodimerize in response to blue light irradiation [207] and thereby reconstituting an active Cas9 protein. When expressed in HEK293T cells, the paCas9 proved effective in inducing targeted genome sequence modifications through both NHEJ and HDR pathways. Conversely, the components dissociated and the genome editing activity has been shown to turn off by simply extinguishing the light source [206].

In the past, it was shown that the catalytic activity of the PvuII restriction endonuclease (REase) could be controlled by a photoswitch involving a derivative from a bifunctional azobenzene [203]. However, unlike “Magnets”, which heterodimerize and activate the paCas9 protein under blue light, the azobenzene-derivative photoswitch deactivates the PvuII REase under blue light and activates it only under illumination by ultraviolet (UV) light (wavelength ~ 365 nm). This system can be turned into a reversible photoswitch as the *trans* isomeric form of azobenzene locks the enzyme in the inactive “off” state, while the *cis* form of azobenzene engages the enzyme into the active “on” state. One important advantage of the photoinducible system is that chemically cross-linked endonuclease in the inactive state can be activated using an external signal light source for DNA-cleavage activity at the specific target sites after being successfully transported into the nucleus of the cell using an appropriate delivery system, such as cationic amphiphilic lipids [208]. One potential concern is that near UV light might be damaging to DNA [209], [210].

Conditional activation of the Cas9 enzyme has also been developed by placing a 4-hydroxytamoxifen (HT)-responsive intein sequence (37R3-2) within the Cas9 ORF, where the intein has been engineered to splice from the host protein when a cell-permeable small ligand (4-HT) is added to the media (Fig. 2f) [211]. In the same study, when the HEK293-GFP cells were treated with 4-HT for 12 h, intein-Cas9 variants in combination with the sgRNAs that target the well-studied *EMX*, *VEGF* and *CLTA* loci exhibited substantially improved specificity compared to that of wild-type Cas9. The presence of 4-HT in the cell culture media increased the on-target modification frequency of the intein-Cas9(S219) variant by 3.4- to 7.3-fold than what was observed and statistically calculated in the absence of 4-HT. However, this system suffers from the reversibility issue in a way that, when the intein splices out of the Cas9 protein, it could not be turned off because the intein cannot be inserted back within the Cas9 ORF [211].

Recently, in order to address the periodic modulation of the Cas9 function, a chemical-inducible CRISPR/Cas9 system was developed, where switching the activity of the Cas9 (or iCas in this case) to both ‘on’ and ‘off’ states were possible [212]. In that study, the authors have shown that a tight spatiotemporal control over the Cas9 protein (iCas9) could be achieved by fusing two hormone-binding domain of the estrogen receptor (ERT2) on each terminus of the Cas9 protein (i.e. (ERT2)_2_–Cas9–(ERT2)_2_). In this configuration, Cas9 cannot enter the nucleus of human cells, thereby preventing the access to the genomic DNA for editing purpose. However, the addition of the ligand 4-HT permits the translocation of the fusion protein into the nucleus [212].

This ligand-based Cas9 activation approach can be used in conjunction with other strategies that are dedicated to reduce off-target issues, such as using paired Cas9 nickases [126], truncated guide RNAs [153], or FokI-dCas9 fusions [213]. In this context, we find that the ligand-dependent intein-based regulation is somewhat analogous to the group II intron ribozyme-based molecular switches that can be promoted to splice at the transcriptional level in order to reconstitute a contiguous active HEase ORF when suitable levels of Mg^+ 2^ are present in the media [184], [185].

Another regulation strategy involves the use of a destabilizing domain (DD) tag (12 kDa, 107 amino acid), which is based on a mutant of the FKBP12 protein (Fig. 2g) [214]. When the DD tag is attached to a protein of interest and expressed as a fusion protein, it leads to the rapid degradation of the protein in the cell by proteasomes. However, a protective effect is observed when the DD's small (750 Da), membrane-permeant ligand (Shield-1) is added to the culture medium. This small ligand reversibly binds to the DD tag and protects the DD-tagged protein from degradation, leading to rapid accumulation of the tagged protein in the cell [215]. Previously, it was shown that linking a modified destabilizing FKBP12 (i.e. DD tag) domain to the amino-terminus of a ZFN protein destabilized the enzyme. A small molecule that blocks the destabilization effect of the amino-terminal domain was used to regulate the ZFN levels and this helped in maintaining higher rates of ZFN-mediated gene targeting while reducing genotoxicity [216]. Recently, Senturk and coworkers have shown that by fusing the FKBP12-derived DD to Cas9 (DD-Cas9), conditional regulation of Cas9 protein stability using DD ligand (Shield-1) could be achieved. Cas9 stability was reversed 2 h following Shield-1 ligand withdrawal from the media; the Cas9 levels were noted to be negligible within 12 h [217].

The various strategies of unifying the split-Cas9 into an active enzyme, or harnessing the splicing reaction of the internal introns in order to yield a functional MN, have been impressive. However, to the best of our knowledge, these “inducible” systems have not been put into any clinical settings. A list of current regulatable genome editing tools has been provided in Supplementary Table 1.

A recent study showed that there are natural inhibitors for CRISPR/Cas9 [218]. These inhibitors can bind to the Cas9 protein and they appear to be encoded by mobile elements and probably have evolved as defense mechanism by phages to counteract the bacterial CRISPR based immune systems. Three families of proteinaceous type of inhibitors have been identified in *Neisseria meningitidis* (Nme) that can potentially be used in human cells as “off” switches against NmeCas9 based genome editing reagents [218]. This could be a seminal study that will lead to further explorations on isolating natural Cas9 inhibitors, and the genes that encode them. These genes that encode Cas9 inhibitors under the control of inducible promoters could be employed as “off” switches in genome editing protocols.

## 4 Alternative Strategies for Developing Regulatable Genome Editing Reagents

Currently, there is a lot of focus on protein-based genome manipulation reagents. However, there are some noteworthy developments in trying to use oligonucleotides as potential alternatives to protein-based genome manipulation tools or as components of such systems (such as the previously discussed TFO nucleases or PNA based application). There would be several advantages of using oligonucleotides such as (a) ease of oligonucleotide synthesis and sequence verification, (b) predictable Watson-Crick base pairing allows for easier design against target sequence and addressing off-target issues, (c) the modular nature of RNA domains/structures permits engineering of multifunctional molecules [219], (d) design of oligonucleotides that target almost any molecule can be achieved by in vitro selection [220], [221], [222], (e) thermally denatured oligonucleotides are generally easier to renature than proteins, (f) some oligonucleotides are functional in the absence of protein factors (additional factors can increase the likelihood of side reactions), and (g) oligonucleotides are less likely to elicit an immune response. Some disadvantages or challenges include: (a) they can be difficult to identify and/or validate target candidates (i.e. not all oligonucleotides can be engineered to be inserted at any position within a sequence), (b) combining different oligonucleotide domains may decrease their efficiency and/or activity [223], (c) can form tertiary interactions, which is currently not fully understood (i.e. off-targeting potentially an issue), (d) in some cases, can be toxic to the cell (e.g. if a ligand is required at beyond physiological concentrations), and (e) prone to nuclease degradation. The best examples for oligonucleotide-based systems used to manipulate gene expression currently are RNAi and self-cleaving hammerhead [224], [225]. These reagents allow for targeted control of gene expression by promoting the removal of specific mRNAs from the cytoplasm. Considerable work is still needed to develop oligonucleotides-based systems for genomic manipulation/editing.

Numerous oligonucleotide molecules are currently studied that could be coopted into regulatory elements at the mRNA level, but only select examples will be mentioned. These will be used to illustrate the potential for oligonucleotides as components of genome editing reagents. More specifically, how oligonucleotides could be incorporated as regulatory elements within protein-based genome editing reagents to refine their activity and accuracy of target site recognition.

### 4.1 The Utility of Hammerhead Ribozymes and Engineered Variants

The hammerhead ribozyme (HHR), first seen in tobacco ringspot virus satellite RNA [226], is an example of small nucleolytic RNA molecules capable of self-cleavage (i.e. ribozymes) [227]. Other autocatalytic (self-cleaving type) small RNA molecules are twister, twister sister, pistol, and hatchet ribozyme [228], [229]. HHRs are composed of a conserved central sequence with three radiating helical domains [230]. Natural HHRs are not true ribozymes as they are only capable of carrying out a single self-cleavage reaction. Synthetic HHRs have been engineered to overcome this by separating the HHR into two components: ribozyme (the part of the HHR which remains unchanged) and substrate (the target sequence that will be cleaved).

Since their discovery in 1986 [226], HHRs have been noted in all domains of life [231] and have been extensively studied and modified. Several aspects of HHRs make them attractive as scaffolds for the development of regulatory switches for genome editing reagents: (a) short sequence (~ 50 nt for an active HHR [232]; enables faster troubleshooting and optimizing, and low cost of synthesis, (b) catalytic activity does not require any protein factors, which can lead to side reactions, (c) can be used as a genome editing tool, and (d) can be designed to cleave two different targets [233]. Some challenges of using HHRs are: (a) the substrate/target needs to be single-stranded in order for the ribozyme to bind, (b) minimal HHRs require Mg^+ 2^ concentrations to be above 10 mM, which is significantly higher than physiological concentrations (~ 0.1 mM) [234], (c) requirement of 5′-UX-3′ sequence at cleavage site, where X can be either A, C, or U [235] limits substrate design (although the limitation is not severe as such sequence is common within a genome).

HHRs are typically used as on/off switches at the mRNA level [236]. They can be integrated in the 5′- or 3′-untranslated region (UTR) in constructs that express genome editing reagents, and self-cleavage could regulate processing of the mRNA. Depending on the organism, insertion of ribozymes in the 5′- or 3′-UTR can evoke different effects. In prokaryotes, a common strategy is to engineer a HHR into the 5′-UTR such that one of the stems (stem I) of the HHR is modified to sequester (through base pairing) the ribosome binding sites (RBS in bacteria) or other features required for initiating translation (i.e. Kozak sequence in eukaryotes). Upon self-cleavage, the ribozyme is removed and the RBS is exposed permitting ribosome access and subsequent protein synthesis. In eukaryotes, using a similar approach to turn on translation would be difficult because self-cleavage of the ribozyme would lead to the removal of the 5′-cap, or 3′-poly(A) tail if the ribozyme if insertion occurred in the 3′-UTR. Both the 5′-cap, and 3′-poly(A) tail, play an essential role in mRNA stability and translation. Although HHRs have been successfully engineered into 5′-UTR [236], the use of this region, in general, can be challenging as the formation of hairpin structures may impede translation [237]. An alternative is the use of the 3′-UTR [238]. Here, self-cleavage is typically used to destabilize the mRNA; ultimately leading to its decay (i.e. ribozyme activity turns off protein expression).

Alternative designs with regards to HHRs are oligonucleotides developed by Erdmann's group [239]. They developed a “mirror-image” hammerhead ribozyme and deoxyribozymes (DNAzymes), termed Spiegelzymes®, enantiomers of the biological d-nucleic acids. The advantage of using L-nucleic acids is that they are less prone to nuclease activity while still being able to interact with d-nucleic acids [240]. This strategy of generating enantiomers or synthetic analogs provides an alternative to standard HHR type molecules, which while more readily accessible, are less stable in an in vivo environment [241].

Another application of the HHR backbone involves the development of temperature-sensitive HHRs. Here, the incorporation of a “RNA thermometer” provides control over HHR activity. By replacing a stem-loop (stem III) of a HHR with a temperature-sensitive hairpin, Saragliadis et al. [242] developed a regulatory element where self-cleavage of a HHR is controlled by changes with temperature; cleavage occurs at lower temperatures but is inhibited at higher temperatures due to denaturing of the temperature-sensitive hairpin required in the formation of a catalytically active HHR. When this element is incorporated within the 5′-UTR region of an mRNA the self-cleavage reaction liberates features of the mRNA needed for initiating the translation of the transcript. The advantage of temperature-based systems is that the switch portion of the regulatory element does not require a ligand (a core requirement for standard riboswitches), which can pose as a challenge as many natural ligands are not long-lived, particularly in an in vivo environment. Although temperature control eliminates the need for a ligand, it can pose as a challenge, especially in mammals, where body temperature is tightly regulated. Thus, the use of such temperature-sensitive hairpins may be limited to prokaryotes and simpler eukaryotes (such as *Saccharomyces*), or in vitro experiments where temperature conditions can be more easily controlled.

### 4.2 Utility of Riboswitches and Allosteric Ribozymes

In many cases, temporal control of protein expression is desired (like components of genome editing tools). Thus, the sole use of a ribozyme is not sufficient as the ribozyme will self-splice once it is properly folded. Therefore, the ribozyme activity needs to be controlled, for example by the integration of a riboswitch element within the ribozyme molecule.

Riboswitches are RNA elements that modulate mRNA expression through binding of a ligand, which is typically a small organic molecule or ion, to its aptamer domain [243]. Ligand binding causes a conformational change in another part of the RNA, referred to as the expression platform, and alters mRNA expression [244]. The effect can either be positive or negative (i.e. promote or inhibit expression). There are currently numerous known riboswitches which can bind to a wide range of ligands including natural and synthetic analogs [243]. Advantages of riboswitches include: (a) no additional proteins are required (which can be toxic and/or deplete vital cell resources), (b) regulation of gene expression is achieved without the use of heterologous gene expression systems, which is characteristic of protein-based systems, and (c) ligand can be administered directly (protein-based systems typically require a transcription effector to be expressed from a plasmid vector).

Recently, several naturally occurring ribozymes have been described that may provide avenues for engineering regulatory switches that could be incorporated into genome editing reagents. Lee et al. [245] noted, in *Clostridium difficile* strain 630, a gene where the expression is regulated by both a riboswitch (aptamer binds c-di-GMP) and a ribozyme (group I intron), which occurs in tandem. In the cyclic di-GMP (c-di-GMP) example, activation of the riboswitch causes a conformational change resulting in splicing of the ribozyme (group I intron) and placement of the ribosome binding site at optimal distance from the start codon. When the ligand (c-di-GMP) is not bound to the aptamer domain, alternative splicing occurs generating a truncated mRNA with no ribosome binding site, which results deficient gene expression. An interesting feature of this riboswitch-ribozyme is that even with correct splicing of the ribozyme, the riboswitch continues to regulate gene expression. This is accomplished by the notion that the RBS remains sequestered in the basal stem of the riboswitch under low ligand concentrations, which inhibits binding of the ribosome. The gene is only expressed when ligand concentrations are high. This allows for tighter control and limiting leaky splicing of the ribozyme. Such tight regulation of gene expression would be desirable in engineering programmable genome editing systems in order to achieve temporal control to reduce off-target activities.

The glmS ribozyme is an example of an oligonucleotide that is both a ribozyme and riboswitch. It demonstrates how the modular nature of RNA can be applied to regulate gene expression. The expression of the *glmS* gene, encoding for glutamine-fructose 6-phosphate transaminase that catalyzes the formation of glucosamine 6-phosphate (GlcN6P), is regulated by binding of the ligand, GlcN6P. The ligand functions as a coenzyme; binding of the ligand provides an amine which participates in general acid-base catalysis [246]. When this element is incorporated into an expression system, self-cleavage of the mRNA leads to non-sense mediated decay. In this example, the control of gene expression is through negative feedback (i.e. binding of the enzyme product negatively influences the expression the enzyme). This strategy can be applied to modulate expression of a gene of interest (such as a component of the genome editing system) by designing a riboswitch–ribozyme element that responds to increasing protein concentration. Thus, protein concentrations can potentially be regulated to remain at relatively low levels. This is important, especially if the genome editing reagent is toxic in high concentrations, associated with off-target activities, or severely affects cell viability.

Another strategy is to incorporate an aptamer domain into the ribozyme, rather than having the ligand-binding and ribozyme components as separate entities (as above examples). Allosteric ribozymes [247], or aptazymes [248], are synthetic ribozymes whose catalytic activity is modulated by ligand binding. Several examples of aptazymes have been successfully synthesized and shown to be functional in both prokaryotic and eukaryotic systems [236], [249], [250]. Again, these types of engineered ribozymes could be part of expression vectors that encode components of genome editing systems.

## 5 Conclusion

Genome editing reagents are being developed and employed at a rapid rate. In order to increase specificity and avoid or reduce toxicity issues due to off-target activities strategies are now being developed to provide temporal control over the DNA-cutting activities of genome editing tools. The review presented a variety of novel approaches that have been employed so far but it also highlights the tremendous potential that is offered by nucleic acid-based regulatory switches that could be incorporated into the expression vectors of genome editing reagents. The development of programmable genome editing tools along with the ability of controlling the temporal and spatial expression of such editing reagents promises to be a very active and challenging research area.

The following is the supplementary data related to this article.

Supplementary Table 1 Current regulatable genome editing tools Supplementary Table 1:

## Competing interests

The authors have declared that no competing interests exist.

## Figures and Tables

**Table 1:** Generalized comparison of various genome engineering tools.

Nuclease platform	MN	ZFN	TALEN	Targetron	CRISPR/Cas
Source	Organellar DNA, Bacteria, Phages	Bacteria, Eukaryotes	Bacteria (*Xanthamonas* sp.)	Organellar DNA, Bacteria, Phages	Bacteria (*Streptococcus* sp.)^a^
Number of component(s)	1	2	2	2	1–2 (depends)^b^
Availability of core components^c^	Restricted	Available	Available	Restricted	Available
Type of recognition	Protein-DNA	Protein-DNA	Protein-DNA	RNA-DNA	RNA-DNA
Recognition site (bp)	18–44^d^	18–36	24–40	14–15	17–23
Double strand break pattern	Staggered cut (4 nt, 3′ overhang)	Staggered cut (4–5 nt, 5′ overhang)	Staggered cut (Heterogeneous overhangs)	Staggered cut^e^	SpCas9 creates blunt ends; Cpf1 creates staggered cut (5′ overhang)
Function	Nuclease, Nickase	Nuclease, Nickase	Nuclease, Nickase	Site-specific bacterial gene disruption^f^	Nuclease, Nickase
Best suited for	Gene editing	Gene knockout, Transcriptional regulation	Gene knockout, Transcriptional regulation	Gene knockout	Gene knockout, Transcriptional regulation, Base editing
Ease of design	Difficult	Difficult; Design of new ZFNs is much easier than MNs	Moderate	Moderate	Easy
Dimerization required	No	Yes	Yes	No	No
Ease of generating large scale libraries	Laborious	Laborious	Moderately laborious	Unknown	Easy
Specificity	High	Low–Moderate	Moderate	Moderate	Low–Moderate^g^
Multiplexing	Low	Low	Moderately high	Low	High
Gene drive	Possible	Unknown	Unknown	Unknown	Possible
Improved/other versions	MegaTEV, MegaTAL	AZP-SNase	Tev-mTALEN	Thermotargetron	Cpf1, eSpCas9
Cost (USD)^h^	4000–5000	5–10,000	< 1000	450–1500	< 100
Targeting constraints	Chromatin compaction	Non-guanosine rich sequence hard to target	5′ targeted base must be thymine for each TALEN monomer	Entry of RNP complex in nucleus difficult	PAM sequence must follow target site
Efficiency/Inefficiency	Small size of MN allows use in a variety of viral vectors	Small size of ZFN expression cassettes allows use in a variety of viral vectors	Large size of each TALEN makes it difficult to pack in viral vectors	Large size of ribonucleoprotein complex makes it difficult for entry into nucleus	Commonly used Cas9 from *S. pyogenes* is large, impose packaging problems in viral vectors i
Methylation sensitive	Yes	Yes	Yes	Unknown	No
First use in human cells	1994	2003	2011	2015	2013
Immunogenicity	Unknown	Low	Unknown	Unknown	Unknown
Vector packaging^j^	Multiple	Multiple	Few	Multiple	Multiple
Size of mRNA transcripts	Short	Short	Long	Short	Long
Mode of ex vivo delivery in animal cells	Electroporation, Viral transduction, Direct injection into zygotes	Electroporation, Lipofection, Viral transduction, Direct injection into zygotes	Electroporation, Lipofection, Viral transduction, Direct injection into zygotes	Electroporation, Lipofection	Electroporation, Lipofection, Viral transduction, Direct injection into zygotes

Source [13], [21], [45], Number of component(s) [80], Availability of core components [80], Type of recognition [81], Recognition site (bp) [42], [49], [51], [55], [57], Double strand break pattern [42], [79], Function [45], [76], [77], [78], [79], [80], Best suited for [13], [45], [162], Ease of design [77], Dimerization required [76], Ease of generating large scale libraries [77], Specificity [86], Multiplexing [77], Gene drive [37], [38], [39], [40], [41], Improved/other versions [59], [103], [108], [117], [124], [155], [161], Cost (USD) [86], Targeting constraints [77], Efficiency/Inefficiency [77], Methylation sensitive [76], [101], First use in human cells [80], Immunogenicity [77], Vector packaging [86], Size of mRNA transcripts [80], Mode of ex vivo delivery in animal cells [77], [87].

a Most widely used Cas9 is from *Streptococcus pyogenes*. However, Cas9 orthologs, such as the smaller Cas9 proteins from *Streptococcus thermophilus* CRISPR1 (ST1), *N. meningitidis* (NM) and the large Cas9 protein from *Treponema denticola* (TD), have shown promising results in genome editing [154].

b 1 (if using a complex guide RNA with Cas9 protein) or 2 (if guide RNA and Cas9 delivered separately) [77].

c Availability of core components refers whether the building blocks are restricted to industry, available through and academic collaboration/purchase, or readily and freely available from not for profit agencies or commercial DNA synthesis [86].

d The range used here, encompasses a number of different MNs and not only LAGs. The largest recognition site of a LAG is ~ 31 bp [94].

e The 3′ hydroxyl group of the group II intron serves as a nucleophile and cleaves just one strand of the DNA homing site. The RNA lariat is reverse spliced into the target site and the endonuclease domain of the assisted protein partner cleaves the complementary DNA strand [57].

f Although compromised activity is observed in eukaryotes and mammalian system due to the suboptimal codon usage, translational repression of the RT, nonsense-mediated decay (NMD) of group II intron-containing RNAs and suboptimal magnesium ion (Mg^+ 2^) concentrations, this RNA-guided endonuclease (RGEN) has shown potential for high site-specific retargeting in prokaryotes by reprogramming the intron EBS [194].

g Recently improved specificity has been reported for eSpCas9 enzyme [161].

h Approximate cost required to generate a single, gene specific candidate reagent [86].

i Short oligonucleotides may be packaged along with guide RNAs into a single adeno-associated virus [154].

j Vector packaging refers to the reagents ability to be packaged and delivered in multiple delivery vehicles. However, the size of TALENs makes them the most restrictive in this regard. To date, only one version (derived from *S. aureus*) of CRISPR/Cas9 can be packaged in an adeno-associated viral vector [86].
