# Supplementary material for: Programmable Genome Editing Tools and their Regulation for Efficient Genome Engineering
Source: Comput Struct Biotechnol J. 2017 Jan 12;15:146–60. doi: 10.1016/j.csbj.2016.12.006 (PMC5279741; doi:10.1016/j.csbj.2016.12.006)
Supplement: Supplementary Table 1 — Current regulatable genome editing tools [file mmc1.docx]

Supplementary Table 1: Current regulatable genome editing tools

| **Nuclease platform** | **Technique used for regulation** | **Effectors** | **Application** | **Comment** | **Refs.** |
| --- | --- | --- | --- | --- | --- |
| MN (PI-SceI)  Year: 2002 | Reversible redox switch: Two pairs of cysteine residues, Cys-64/Cys-344 and Cys-67/Cys-365 inserted separately into DNA- binding loop. During reduced state, disulphide bonds break and the loop free for binding DNA substrate (i.e. active form of enzyme). During oxidized state, DNA-binding loop closed due to formation of disulphide bonds (i.e. inactive form of enzyme). | Inductor = 10 mM Dithiothreotol (DTT)  Antagonizer = Dialysis for removal of DTT /adding oxidizing agents | *In vitro* control of PI-SceI activity.  The activity of the protein in the oxidized state is decreased more than 30-fold. | This molecular switch technique is not suitable for *in vivo* studies as redox potentials can damage cells and disrupt other cellular proteins. | 182 |
| MN (I-CthI)  Year: 2016 | Ribozyme (either group II A or II B intron) inserted within the ORF of I-CthI. Splicing of these introns was triggered by spiking the bacterial growth media with metal ions. Removal of group II introns reconstituted a continuous ORF thereby resulted in the expression of an active endonuclease. | Inductor = 5 mM upto 10 mM  MgCl_2_ initiated splicing  Antagonizer = 10 µM CoCl_2_ prevented splicing | Developed as a proof-of-concept study. Both *in vivo* and *in vitro* attenuation of the endonuclease expression have been shown to be possible. | This study may be extended to regulate expression of other DNA-cutting enzymes (Cas9) and DNA-binding proteins like transcription factors; may promote shift in metabolic processes. | 184, 185 |
| REase (PvuII)  Year:  2010 | A bifunctional azobenzene derivative was used to cross-link two suitably placed cysteine residues of PvuII enzyme. The *trans* isomeric form of the azobenzene resulted in locking the enzyme in the inactive “off” state (when illuminated with blue light), while the *cis* form of the azobenzene resulted in the active “on” state (when illuminated with UV). | Inductor = UV light (~365 nm)  *trans* 🡪 *cis* transition (active enzyme)  Antagonizer = Blue light  *cis*🡪 *trans* transition (inactive enzyme) | Upon UV induction, the enzyme exhibited 16-fold increase in DNA-cleavage activity. | Increase in the *cis*/*trans* effect which will directly affect the enzymatic activity may be obtained by combination of several cross-links and introduction of additional amino acid substitutions. | 203 |
| CRISPR/Cas9  Year:  2015 | Split-Cas9 protein where the nuclease lobe and the α-helical lobe were expressed as two separate polypeptides. In the presence of a single guide RNA (sgRNA), the two modules interacted and combined hence, restoring the activity of a full-length Cas9. | A target sequence- specific sgRNA promoted heterodimerization of the α-helical and the nuclease lobe of split-Cas9 enzyme | Targeted the *EMX1* locus in HEK293T cell. When activated, the reconstituted enzyme generated indels with efficiencies from 0.6%-2%. | Split-Cas9 showed reduced level relative to wild type Cas9. This system could be rendered deactivate through the removal of hairpins at the 3′ end of sgRNA. | 199 |
| CRISPR/Cas9  Year: 2015 | Split-Cas9 protein where C- and N-terminal fragments were fused to FK506 binding protein 12 (FKBP), and FKBP rapamycin binding (FRB) domain of the mTOR. Nuclear localization signals were tagged for spatial sequestration of these split fragments inside the cell. | 200 nM rapamycin activated dimerization of the split enzyme and allowed trafficking of Cas9 towards nucleus for inducible gene targeting | On-target indel frequency in HEK293FT cells was ~43% after 12 days treatment with rapamycin compared to ~95% for wild type Cas9. | This system was also used to modulate transcription of various genes.  Abscisic acid or Gibberellin-sensing domains may also be employed. | 201 |
| CRISPR/Cas9  Year:  2015 | Cas9 enzyme was cloned downstream to a tetracycline (doxycycline)-inducible promoter, TRE^3G^ within the TRE3G-GFP-IRES targeting vector. Also, U6 promoter and guide RNA pairs were inserted upstream of the TRE^3G^ promoter site. | Inductor = 1 mg/mL doxycycline (dox) | The dox inducible Cas9 system was transfected in mice KH2 embryonic stem cell targeting *CR8*, *Apc* and *Trp53* genes. | Even though strong induction of the TRE^3G^ promoter in the intestine, skin, and thymus is observed, inducible mutagenesis in wider range of tissues may be possible by using alternate TRE promoters. | 200 |
| CRISPR/Cas9  Year:  2015 | A 4-hydroxytamoxifen (HT)-responsive intein sequence (37R3-2) was placed within the Cas9 ORF where the intein has been engineered to splice from the host protein when a cell-permeable small ligand is added to the media. | Inductor = 1 µM 4- hydroxytamoxifen | Targeted the genomic *EGFP*  locus in HEK293-GFP cells. Also targeted *EMX*, *VEGF*, and *CLTA* loci for genome editing. | Genome editing in the presence of 4- hydroxytamoxifen  was 3.4- to 7.3-fold higher than in the absence of 4- hydroxytamoxifen. | 211 |
| CRISPR/Cas9  Year:  2015 | Photoactivatable Cas9 (paCas9) where each split-Cas9 fragment was appended to photoinducible dimerization protein domains termed “Magnets” (pMag and nMag). These elements dimerize in the presence of blue light, thereby reconstituting a complete, active Cas9 protein. | Inductor = Blue light irradiation  Antagonizer = Extinguishing the light source (dark) | The paCas9 proved effective in inducing targeted genome sequence modifications in human embryonic kidney 293T cells. | Inactive state of the enzyme can be activated using an external signal (light) for DNA-cleavage activity after being transported into the nucleus. | 206, 207 |
| CRISPR/Cas9  Year:  2015 | Fusing the FKBP12-derived destabilization domain (DD) to Cas9 (DD-Cas9), conditional regulation of Cas9 protein stability using DD ligand (Shield-1) could be attained. Actually, this small ligand reversibly binds to the DD tag and protects the DD-tagged protein from degradation thereby, rapid accumulation of the tagged protein in the cell. | Inductor = 200 nM Shield-1 (DD ligand) | Several genes involved in oncogenic transformation, DNA damage responses, DNA replication/repair, and mitochondria metabolism were targeted with DD-Cas9 fusion protein. | The regulation of the DD-Cas9 expression is not dependent on its mRNA expression. This allows the DD-Cas9 to be co-expressed with any other gene of interest (from the same promoter) without the need for co-modification. | 217 |
